# Naked Mole-Rat Cortex Maintains Reactive Oxygen Species Homeostasis During *In Vitro* Hypoxia or Ischemia and Reperfusion

**DOI:** 10.2174/1570159X20666220327220929

**Published:** 2023-05-12

**Authors:** Liam Eaton, Tina Wang, Maria Roy, Matthew E. Pamenter

**Affiliations:** 1 Department of Biology, University of Ottawa, Ottawa, ON, Canada;; 2 University of Ottawa Brain and Mind Research Institute, Ottawa, ON, Canada

**Keywords:** Anoxia, sodium cyanide, H_2_O_2_, superoxide, mitochondria, electron transport system

## Abstract

Neuronal injury during acute hypoxia, ischemia, and following reperfusion are partially attributable to oxidative damage caused by deleterious fluctuations of reactive oxygen species (ROS). In particular, mitochondrial superoxide (O_2^•^_^-^) production is believed to upsurge during low-oxygen conditions and also following reperfusion, before being dismutated to H_2_O_2_ and released into the cell. However, disruptions of redox homeostasis may be beneficially attenuated in the brain of hypoxia-tolerant species, such as the naked mole-rat (NMR, *Heterocephalus glaber*). As such, we hypothesized that ROS homeostasis is better maintained in the brain of NMRs during severe hypoxic/ischemic insults and following reperfusion. We predicted that NMR brain would not exhibit substantial fluctuations in ROS during hypoxia or reoxygenation, unlike previous reports from hypoxia-intolerant mouse brain. To test this hypothesis, we measured cortical ROS flux using corrected total cell fluorescence measurements from live brain slices loaded with the MitoSOX red superoxide (O_2^•^_^-^) indicator or chloromethyl 2’,7’-dichlorodihydrofluorescein diacetate (CM-H_2_-DCFDA; which fluoresces with whole-cell hydrogen peroxide (H_2_O_2__)_ production) during various low-oxygen treatments, exogenous oxidative stress, and reperfusion. We found that NMR cortex maintained ROS homeostasis during low-oxygen conditions, while mouse cortex exhibited a ~40% increase and a ~30% decrease in mitochondrial O_2^•^_^-^ and cellular H_2_O_2_ production, respectively. Mitochondrial ROS homeostasis in NMRs was only disrupted following sodium cyanide application, which was similarly observed in mice. Our results suggest that NMRs have evolved strategies to maintain ROS homeostasis during acute bouts of hypoxia and reoxygenation, potentially as an adaptation to life in an intermittently hypoxic environment.

## 1 INTRODUCTION

Reactive oxygen species (ROS) are potent cellular second messengers involved in the regulation of propagating growth factors, immune responses, hypoxia-inducible factors, and protein function, among other roles [1-5]. As such, ROS are tightly regulated when the cell is in homeostasis [6-8]. However, an upsurge in ROS can be deleterious [9], resulting in dysregulation of cellular functions and/or oxidative damage to proteins, DNA, and lipids [6, 10, 11], and may also promote apoptotic cell death [12].

The mitochondrial electron transport system (ETS) is the major producer of ROS in most cells, with 0.25-4% of all oxygen consumed for the process of oxidative phosphorylation leaking out of the ETS to generate highly reactive superoxide anions (O_2^•^_^-^) [13]. Due to oxygen’s central involvement in ROS generation, it is not surprising that changes in environmental oxygen availability impact ROS homeostasis. Indeed, elevated ROS production has been observed across a variety of species, tissues, and experimental preparations during and following exposure to various hypoxic protocols [14-16], oxygen-glucose deprivation (OGD; a common *in vitro* model of ischemia) [17-19], and chemically-induced anoxia (with cyanide: NaCN) [18].

The hypoxia-driven increase in ROS production is usually attributed to mitochondria. Specifically, oxygen is required by complex IV for this enzyme to accept electrons from complex III through cytochrome C [20]. When oxygen is not available, this reaction cannot proceed in the forward direction, and instead, the flow of electrons along the ETS reverses. When this occurs, complex I activity slows, eventually reverses, and begins to produce greater amounts of ROS due to a reduced coenzyme Q pool and the absence of NADH [21, 22]. Importantly, complex II activity also begins to reverse, which results in succinate accumulation proportional to the severity and duration of hypoxic (or ischemic) exposure [21, 23, 24]. Upon reoxygenation, the ETS once again flows in the forward direction, causing the accumulated succinate pool to be rapidly dismutated by succinate dehydrogenase, which floods the coenzyme Q pool with electrons. This results in an upsurge of ROS production through complex I and is believed to be a major source of deleterious ROS generation during reperfusion injury [23, 25].

Given the impact of environmental hypoxia on redox homeostasis, and the potential for dysregulation of cellular pathways and cell damage and death when ROS is imbalanced, it is intriguing to consider the *in vivo* conditions experienced by the brain of animals that live in intermittent or severe hypoxia in their natural environment. If repeated bouts of hypoxia are deleterious, then generations of animals exposed to lifetimes of such conditions would presumably adapt to either limit fluctuations in cellular ROS generation during periods of hypoxia or/and have an enhanced ability to scavenge ROS relative to that of hypoxia-intolerant species. For example, in the anoxia-tolerant western painted turtle cortex, ROS production decreases in anoxic conditions, and bursts of ROS are not observed during reoxygenation [26-28]. However, little is known regarding ROS management in hypoxia-tolerant mammal brain during hypoxia and following reoxygenation.

Naked mole-rats (*Heterocephalus glaber*; NMRs) are an example of a terrestrial mammal that inhabits an intermittently hypoxic environment. Specifically, NMRs putatively experience intermittent hypoxia in their burrows, with relatively harsher hypoxic conditions found in more densely populated burrow regions [29-31]. Indeed, NMRs are robustly hypoxia-tolerant for an adult mammal, remaining active in several hours of severe hypoxia (3% O_2_) [32, 33], and surviving for up to 18 minutes in anoxia while remaining active for 60 seconds [32, 34]. *Ex vivo,* NMR brains are also tolerant to hypoxia, with an improved ability to maintain synaptic transmission, and delayed onset of excitotoxic cell death during prolonged hypoxic exposure compared to mice [35].

Recent research has begun to elucidate functional responses in NMR mitochondria that may help to maintain ROS homeostasis or minimize deleterious ROS bursts during periods of fluctuating O_2_ availability. For example, following 4 hrs of acute *in vivo* hypoxia, total ETS flux reversibly decreases by up to 85% in NMR brain mitochondria, and proton leak decreases concomitantly [36]. This decrease in mitochondrial flux matches the whole body's decrease in metabolic rate in these hypoxic conditions. Furthermore, although the metabolic rate has not been directly measured in hypoxic NMR brain, energetically-demanding Na^+^/K^+^-ATPase activity decreases in the cortex following similar treatments [37], while nicotinate and nicotinamide metabolism use is altered, which indicates that NMR brain mitochondria decrease oxidative phosphorylation during hypoxia [38], while brain [ATP] is maintained [39]. Together, these data suggest that the NMR brain achieves a hypometabolic state *in vivo*, which is expected to decrease ROS generation because the production of ROS from mitochondria is linked to the rate and efficiency of cellular metabolism.

To date, ROS production has not been measured in hypoxic NMR brain; however, it is notable that ROS homeostasis is maintained in NMR skeletal muscle following acute *in vivo* hypoxia [40], and NO homeostasis is maintained in NMR brain during acute *in vitro* hypoxia or ischemia [41]. Furthermore, NMR brain homogenates exposed to short periods of ischemia and ischemia-reperfusion *in vitro* exhibit smaller increases in electron leak and H_2_O_2_ generation than mouse brain homogenates [42]. Finally, NMR cardiac and skeletal muscles have five- and two-fold greater capacity, respectively, to consume H_2_O_2_ than mice [43, 44], which hints at the potential for enhanced ROS scavenging capacity in other NMR tissues (*e.g.*, brain), which may help to buffer fluctuations in ROS generation during periods of intermittent hypoxia.

Taken together, these studies have revealed several interesting modifications that may minimize ROS generation, maximize ROS scavenging, and contribute to the tolerance of the NMR brain to hypoxia and ischemia. Thus, NMRs provide a compelling model in which to test the hypothesis that ROS homeostasis is better maintained in the brain of hypoxia-adapted mammals. Unfortunately, studies examining ROS production and consumption and the impacts of ROS imbalance during periods of hypoxia or ischemia are lacking in the NMR brain. To begin addressing this knowledge gap, we used fluorescence microscopy to measure the generation of mitochondrial O_2^•^_^-^ and cellular hydrogen peroxide (H_2_O_2_, as a proxy for whole-cell ROS generation), from NMR and mouse cortical brain slices exposed acutely to 20 minutes of normoxia (control), hypoxia (1% O_2_), ischemia (OGD), anoxia (NaCN), or exogenous H_2_O_2_ addition (to examine the impact of exogenous ROS addition on cellular redox homeostasis).

## 2 MATERIALS AND METHODS

### 2.1 Animals

NMRs were group-housed in interconnected multi-cage systems at 30**°**C and 21% O_2_ in 50% humidity with a 12L:12D light cycle. Animals were fed fresh tubers, vegetables, fruit, and Pronutrocereal supplement *ad libitum*. CD-1 mice were obtained from Charles River and were housed at room temperature under a 12L:12D light cycle and fed rodent chow *ad libitum*. Animals were not fasted prior to experimental trials. All experimental procedures were approved by the University of Ottawa Animal Care Committee (protocol #3444) in accordance with the Animals for Research Act and by the Canadian Council on Animal Care.

### 2.2 Tissue Preparation and Experimental Design

A total of 28 adult subordinate NMRs (1-2 years old) and 19 male adult mice (10-16 weeks old) were euthanized with cervical dislocation followed by decapitation. Brains were then rapidly removed on ice and 300 μm thick transverse sections were sectioned with a vibratome in ice-cold oxygenated (95% O_2_, 5% CO_2_) N-methyl-D-glucamine (NMDG)-based artificial cerebral spinal fluid (ACSF) containing (in mM): NMDG 120, NaH_2_PO_4_ 1.25, MgCl_2_ 7, CaCl_2_ 1, KCl 2.5, NaHCO_3_ 25, d-glucose 20, Na-pyruvate 2.4, and Na-ascorbate 1.3; pH 7.3. The resulting sections were then incubated at 28°C for 30 minutes and then again at room temperature for 30 minutes in ACSF containing (in mM): NaCl 126, NaH_2_PO_4_ 1.25, MgCl_2_ 1.5, CaCl_2_ 2, KCl 2.5, NaHCO_3_ 26, and d-glucose 10; pH 7.3. Individual slices were pre-loaded with 8 μl Cremophor EL solution (0.5% in DMSO) in aerated 3mL ACSF baths for 5 minutes and then loaded with specific fluorophores for 30 minutes at room temperature in oxygenated ACSF.

We used 2.5 μM CM-H_2_-DCFDA (Invitrogen, California, USA) and 2.5 μM MitoSOX Red Superoxide Indicator (Invitrogen) to measure fluctuations in the production of H_2_O_2_ and mitochondrial O_2^•^_^-^, respectively. Importantly, O_2^•^_^-^ is rapidly converted to H_2_O_2_ in the cytosol and so cellular H_2_O_2_ measurements are more likely to be indicative of total ROS changes within a given tissue. Following incubation, the excess dye was rinsed off and slices were placed in a chamber perfused with ACSF at a flow rate of ~5 mL/min. Experiments consisted of 10 minutes equilibration period after which experiments commenced with 10 minutes of normoxic perfusion period, followed by 20 minutes of the treatment period, and finally 20 minutes of normoxic ACSF reperfusion. Control trials did not have any perfusion change during the 60-minute treatment period. Hypoxic perfusion was achieved by aerating ACSF with a 95% N_2_, 5% CO_2_ gas mixture in a second reservoir. OGD was achieved using ACSF containing a sucrose equimolar replacement for glucose and bubbled with 95% N_2_ and 5% CO_2_. Chemically-induced anoxia was achieved by applying ACSF containing 3 mM NaCN. To examine the impact of exogenous ROS addition on cellular redox homeostasis, ACSF containing 250 μM H_2_O_2_ was applied.

### 2.3 Fluorescence Microscopy

Fluorescence was measured and quantified from time-course images using Image-J (NIH, Bethesda, USA) to determine fluctuations in H_2_O_2_ and mitochondrial O_2^•^_^-^ production in cortex over time and between treatments. Images were taken with a Zeiss Axio Examiner Z1 microscope at 20x magnification every 2 minutes for 60 minutes. When imaging with DCF dye, an LED light source with a wavelength of 470 nm was used in conjunction with a 38HE filter containing emission wavelengths between 500 nm-550 nm (Zeiss, Axio Examiner Z1). When imaging with MitoSOX red dye, an LED light source with a wavelength of 365 nm was used in conjunction with a 90HE filter containing emission wavelengths ranging 410-440 nm, 499-529 nm, 579-604 nm, and 659-759 nm (Zeiss, Axio Examiner Z1).

### 2.4 Data Collection and Statistical Analysis

Regions of interest were assigned to cortical neurons, as identified morphologically by visual examination [45], and fluorescence was quantified. Following experiments, data were slope-corrected and normalized to baseline measurements collected in the first 10 minutes of each experiment. Repeated measures of two-way ANOVAs with Holm-Šídák multiple comparisons tests were used to analyse differences in fluorescence between species and initial normoxic exposure. Values are reported as mean ± SEM. All statistical analyses were performed using GraphPad Prism 9 (GraphPad Prism, La Jolla, CA, USA), with a significance level of *p <* 0.05.

## 3 RESULTS

**Fig. (1):**
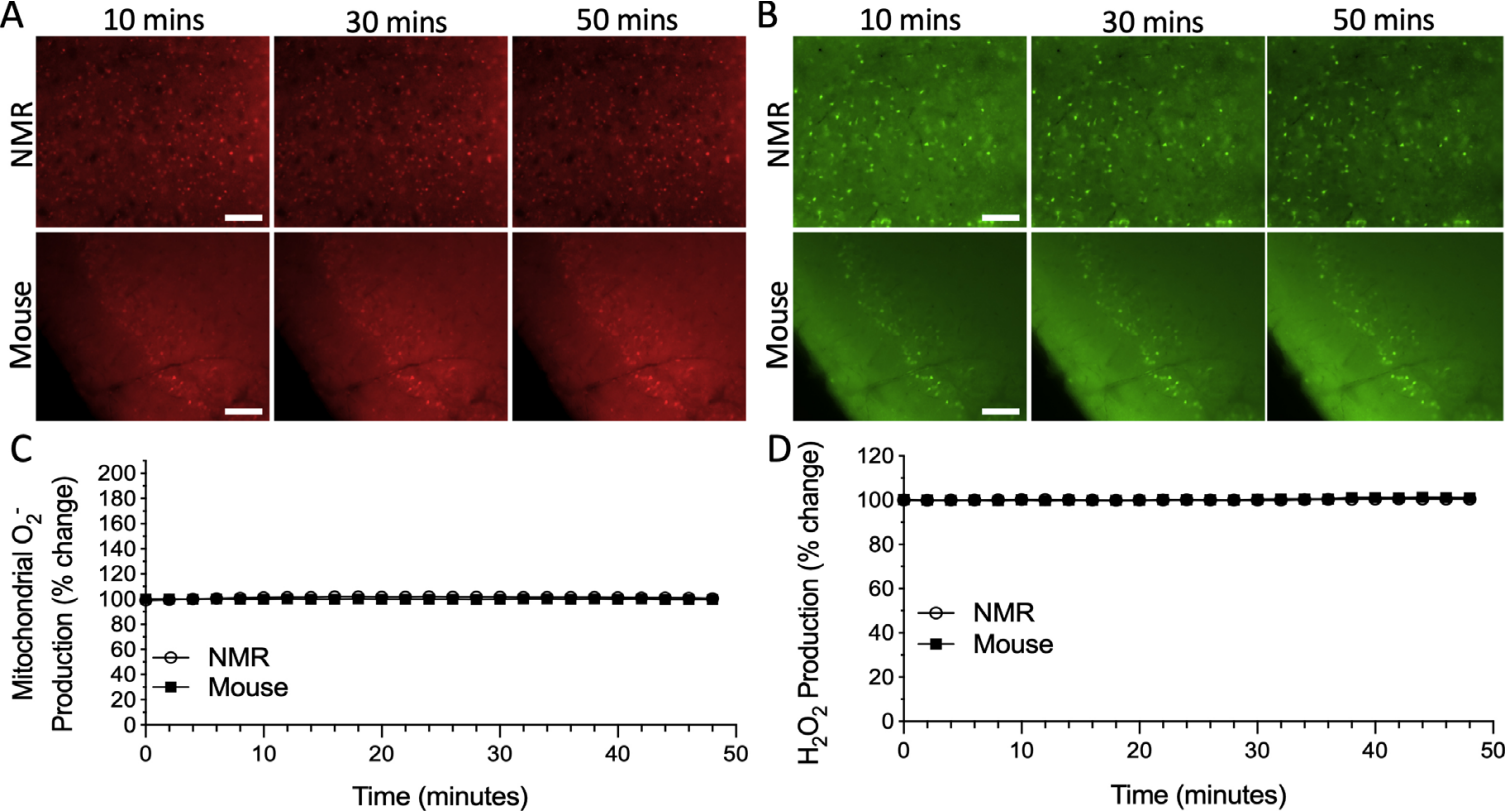
Reactive oxygen species (ROS) production does not fluctuate during sustained normoxic conditions in the cortex of naked mole-rats (NMRs) and mice. Fluorescent time-lapse images of cortical slices loaded with **A**) the O_2^•^_^-^ sensitive mitochondrial fluorophore MitoSOX Red, and **B**) the H_2_O_2_ sensitive fluorophore CM-H_2_-DCFDA. Scale bar = 100 μm. Mean traces of **C**) mitochondrial O_2^•^_^-^ production and **D**) H_2_O_2_ production in the cortex of NMRs (*n* = 11 slices from 9 animals, and 17 slices from 14 animals, respectively) and mice (*n* = 4 slices from 4 animals, and 9 slices from 7 animals, respectively). Individual trials were slope-corrected and normalized based on the initial 10 minutes prior to treatment application. Data are mean ± SEM.

We used fluorophores to measure fluctuations in the production of mitochondrial O_2^•^_^-^ and whole-cell H_2_O_2_ in the cortex of mice and NMRs. In normoxic control experiments, we observed that mitochondrial O_2^•^_^-^ (Fig. **1b**, *n* = 4 slices from 4 mice and 11 slices from 9 NMRs, F_24, 325_ = 0.2640, ns) and whole-cell H_2_O_2_ (Fig. **1d**, *n* = 9 slices from 7 mice and 17 slices from 14 NMRs, F_24, 600_ = 0.3686, ns) were stable in cortical slices from both study species.

### 3.1 ROS Homeostasis is Maintained during Hypoxia and Reoxygenation in NMR but not Mouse Cortex

**Fig. (2):**
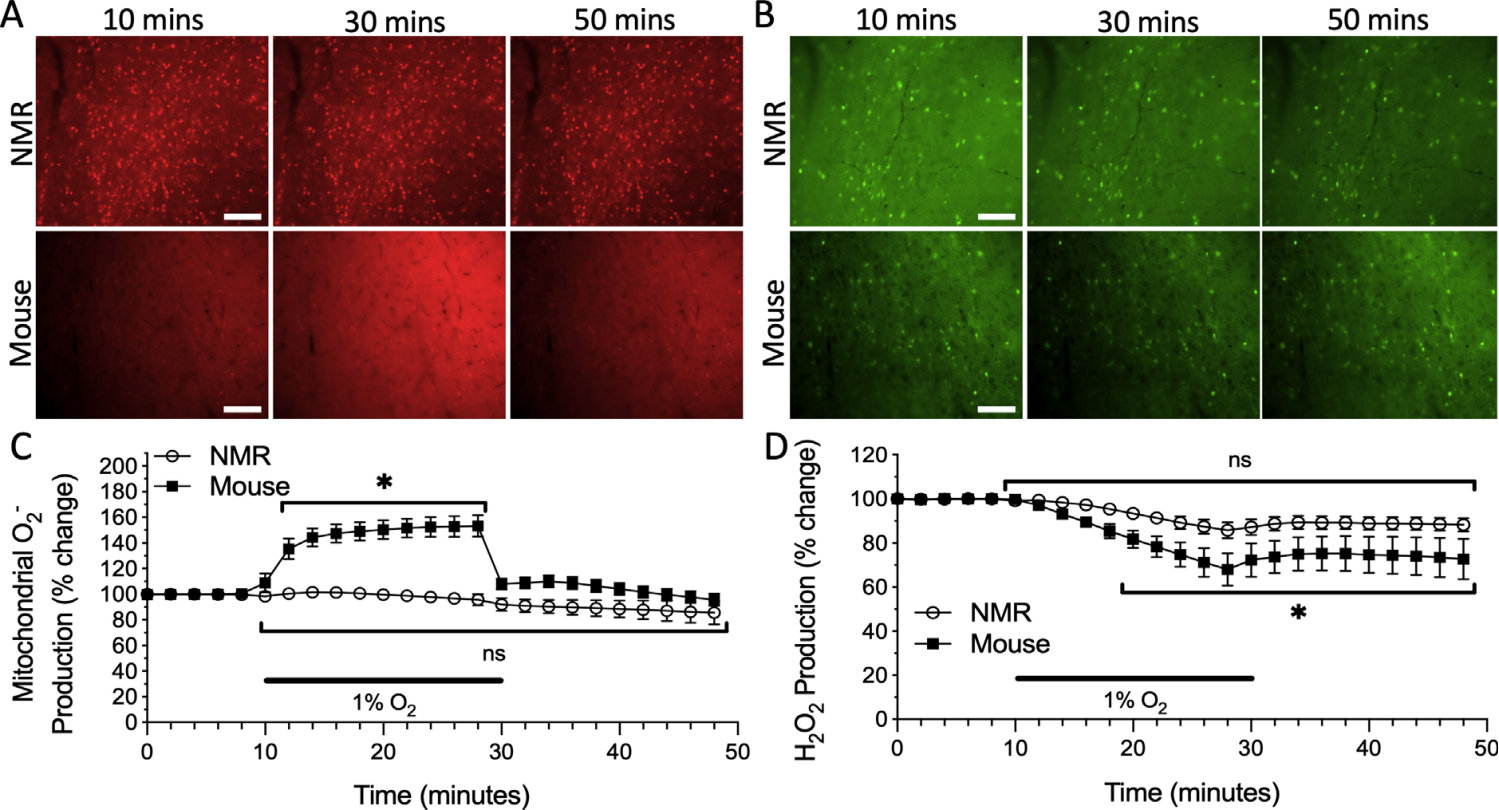
ROS production fluctuates during hypoxic (<1% O_2_) exposure in the cortex of mice, but not NMRs. Fluorescent time-lapse images of cortical slices loaded with **A**) the O_2^•^_^-^ sensitive mitochondrial fluorophore MitoSOX Red, and **B**) the H_2_O_2_ sensitive fluorophore CM-H_2_-DCFDA during initial normoxic conditions, hypoxic treatment, and re-oxygenation. Scale bar = 100 μm. Mean traces of **C**) mitochondrial O_2^•^_^-^ production and **D**) H_2_O_2_ production in the cortex of NMRs (*n* = 7 slices from 6 animals, and 14 slices from 12 animals, respectively) and mice (*n* = 6 slices from 3 animals, and 11 slices from 8 animals, respectively). Individual trials were slope-corrected and normalized based on the initial 10 minutes prior to treatment application. The black bar indicates hypoxic treatment application. Asterisk indicates a significant difference in relation to the initial normoxic value (p < 0.05; Two-way ANOVA with Holm-Šídák multiple comparisons test). Data are mean ± SEM.

Next, we tested the effects of hypoxic exposure and subsequent reoxygenation on ROS production in mouse and NMR cortex and found a significant interaction between time and species (F_24, 275_ = 8.081, p = 0.0001). In the mouse cortex, a sudden and significant increase in mitochondrial O_2^•^_^-^ production was observed during hypoxia, resulting in a 53% increase in signal compared to initial normoxic conditions. Following re-oxygenation, mitochondrial O_2^•^_^-^ production rapidly returned to levels that were not statistically different from control normoxic conditions (Fig. **2b**, *n* = 6 slices from 3 animals, p < 0.0001). In the NMR cortex, mitochondrial O_2^•^_^-^ production did not fluctuate during the hypoxic treatment period or upon re-oxygenation (Fig. **2b**, *n* = 7 slices from 6 animals, p = 0.6078).

Interestingly, there was no statistical effect for the interaction between time and species for H_2_O_2_ production within hypoxia and reoxygenation treatments (F_24, 575_ = 1.277, p = 0.1713); however, there was a time effect (F_24, 575_ = 8.515, p < 0.0001). In contrast to mitochondrial O_2^•^_^-^ production, hypoxic exposure in the mouse cortex resulted in a gradual, but significant, decrease in H_2_O_2_ production to approximately 68% of initial normoxic levels by the end of the treatment period. H_2_O_2_ production remained significantly lowered by 74% of the initial normoxic signal following re-oxygenation (Fig. **2d**, *n* = 11 slices from 8 animals, p < 0.0001). A similar but non-significant trend was observed in the NMR cortex, where H_2_O_2_ production decreased to 87% of initial normoxic levels during hypoxia and remained stable during reoxygenation (Fig. **2d**, *n* = 14 slices from 12 animals, p = 0.19).

### 3.2 ROS Homeostasis is Maintained during Ischemia and Reoxygenation in NMR but not Mouse Cortex

**Fig. (3):**
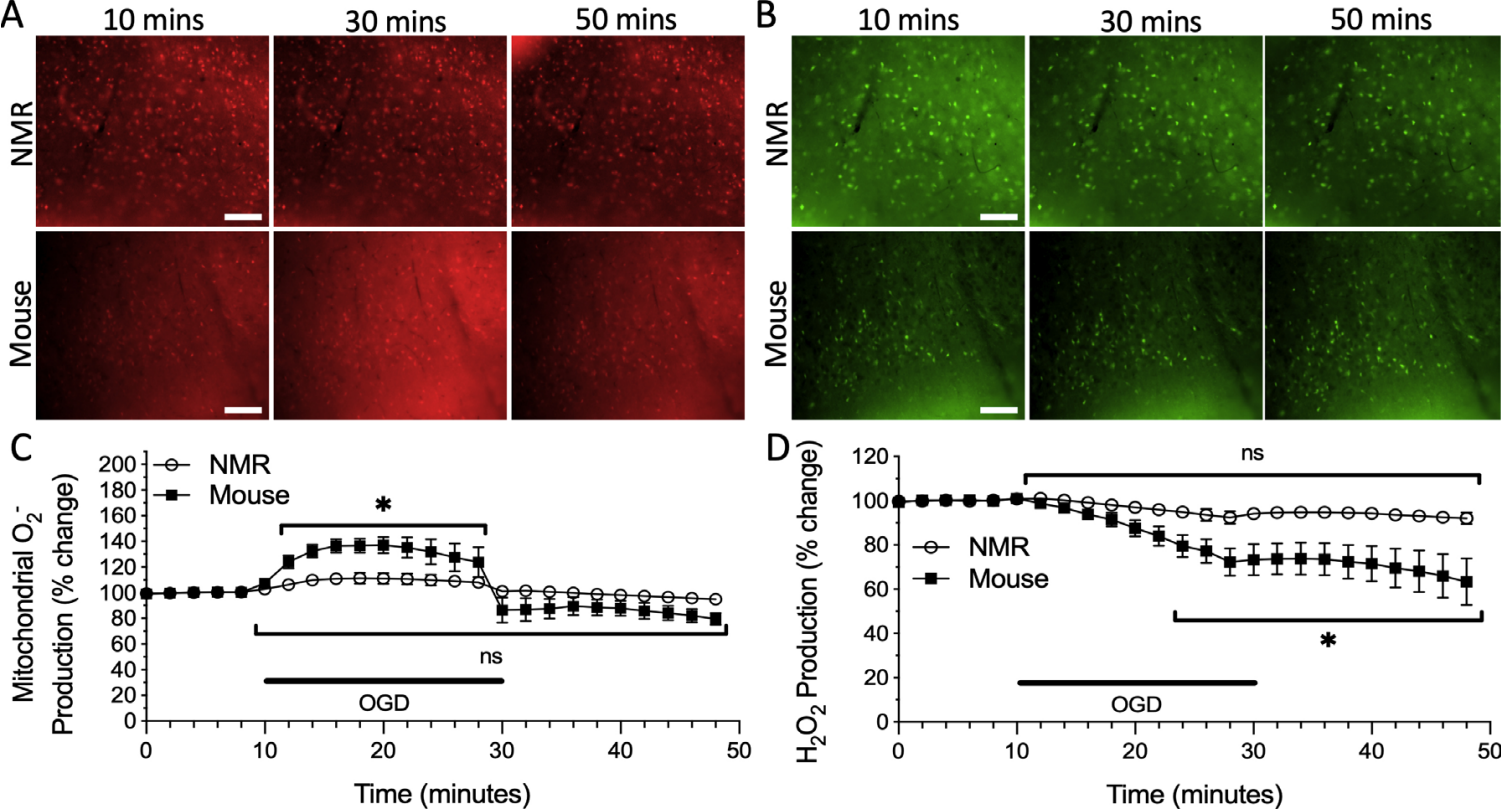
ROS production fluctuates during oxygen-glucose deprivation (OGD) in the cortex of mice, but not NMRs. Fluorescent time-lapse images of cortical slices loaded with **A**) the O_2^•^_^-^ sensitive mitochondrial fluorophore MitoSOX Red, and **B**) the H_2_O_2_ sensitive fluorophore CM-H_2_-DCFDA during initial normoxic conditions, OGD, and re-oxygenation. Scale bar = 100 μm. Mean traces of **C**) mitochondrial O_2^•^_^-^ production and **D**) H_2_O_2_ production in the cortex of NMRs (*n* = 10 slices from 7 animals, and 13 slices from 11 animals, respectively) and mice (*n* = 5 slices from 4 animals, and 7 slices from 6 animals, respectively). Individual trials were slope-corrected and normalized based on the initial 10 minutes prior to treatment application. The black bar indicates the OGD treatment application. Asterisk indicates a significant difference in relation to the initial normoxic value (p < 0.05; Two-way ANOVA with Holm-Šídák multiple comparisons test). Data are mean ± SEM.

We then tested the effect of OGD treatment on cortical ROS production. OGD is a common *in vitro* ischemic mimic and is more challenging than hypoxic treatment alone [17–19]. Nonetheless, the effects of OGD on mitochondrial O_2^•^_^-^ production for both species were similar to those observed during hypoxia, such that a significant interaction between time and species was observed (F_24, 325_ = 7.088, p < 0.0001). Specifically, in the mouse cortex, mitochondrial O_2^•^_^-^ production suddenly and significantly increased by 37% during the treatment period and then rapidly returned to levels similar to initial normoxic conditions upon re-oxygenation (Fig. **3b**, *n* = 5 slices from 4 animals, p < 0.0001). Conversely, mitochondrial O_2^•^_^-^ production did not fluctuate significantly during the OGD treatment period or upon re-oxygenation in NMRs (Fig. **3b**, *n* = 10 slices from 7 animals, p = 0.3032).

Once again, there was a significant interaction between time and species for H_2_O_2_ production during OGD treatment trials (F_24, 450_ = 4.531, p < 0.0001). As in hypoxic experiments, OGD in the mouse cortex resulted in a significant decrease in H_2_O_2_ production to 73% of the initial signal, but this rate continued to decrease to 63% of the initial normoxic signal following re-oxygenation (Fig. **3d**, *n* = 7 slices from 6 animals, p < 0.0001). Again, H_2_O_2_ production in the NMR cortex did not significantly change with OGD or reoxygenation (Fig. **3d**, *n* = 13 slices from 11 animals, p = 0.7622).

### 3.3 Chemical Anoxia Disrupts ROS Homeostasis in both NMR and Mouse Cortex

**Fig. (4):**
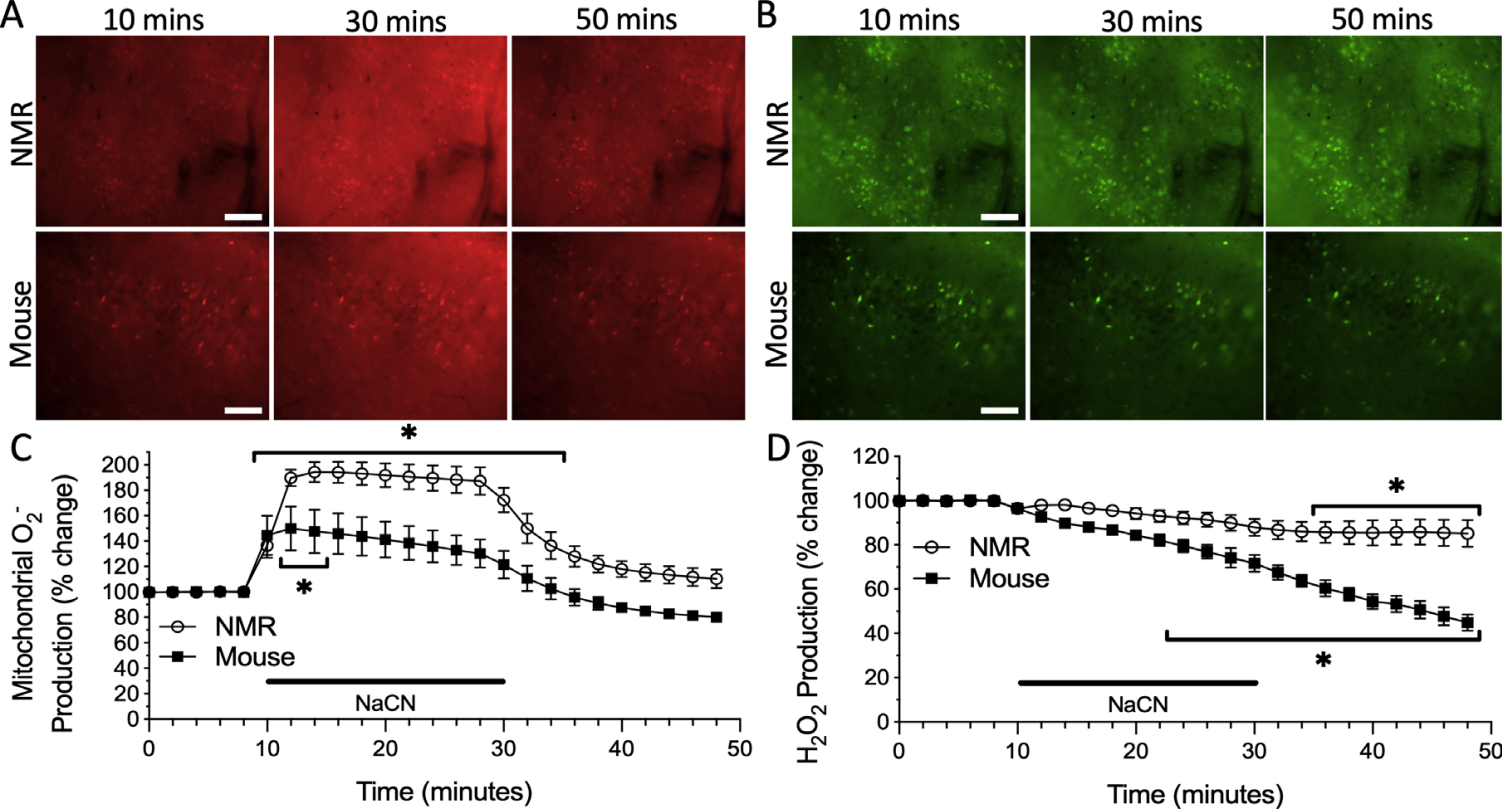
ROS production fluctuates during chemically induced anoxia (3 mM NaCN) exposure in the cortex of mice and NMRs. Fluorescent time-lapse images of cortical slices loaded with **A**) the O_2^•^_^-^ sensitive mitochondrial fluorophore MitoSOX Red, and **B**) the H_2_O_2_ sensitive fluorophore CM-H_2_-DCFDA during initial normoxic conditions, NaCN application, and washout. Scale bar = 100 μm. Mean traces of **C**) mitochondrial O_2^•^_^-^ production and **D**) H_2_O_2_ production in the cortex of NMRs (*n* = 6 slices from 3 animals for both fluorophores) and mice (*n* = 3 slices from 2 animals, and 3 slices from 3 animals, respectively). Individual trials were slope-corrected and normalized based on the initial 10 minutes prior to treatment application. The black bar indicates the NaCN treatment application. Asterisk indicates a significant difference in relation to the initial normoxic value (p < 0.05; Two-way ANOVA with Holm-Šídák multiple comparisons test). Data are mean ± SEM.

Next, we treated slices with ACSF containing 3 mM NaCN to chemically induce anoxia through inhibition of complex IV [18], which yielded a significant interaction between time and species (F_24, 175_ = 2.459, p = 0.0004). In the mouse cortex exposed to NaCN, mitochondrial O_2^•^_^-^ production suddenly and significantly increased by 49% before gradually returning to levels slightly lower than initial normoxic conditions following re-oxygenation (Fig. **4b**, *n* = 3 slices from 2 animals, p = 0.0303). However, unlike in the hypoxic and ischemic experimental groups, the NMR cortex also exhibited a significant upsurge in mitochondrial O_2^•^_^-^ production of nearly 94% with NaCN exposure. After the washout period, NMR mitochondrial O_2^•^_^-^ production gradually decreased to levels just above initial normoxic conditions (Fig. **4b**, *n* = 6 slices from 3 animals, p < 0.0001).

A two-way ANOVA revealed a significant interaction between time and species for H_2_O_2_ production during NaCN application trials (F_24, 175_ = 5.337, p < 0.0001). In the mouse cortex, H_2_O_2_ production significantly decreased to 72% of its initial normoxic value by the end of the NaCN treatment period, but further decreased to 45% of this initial value following washout (Fig. **4d**, *n* = 3 slices from 3 animals, p < 0.0001). This trend was observed in the NMR cortex to a lesser extent but was only significantly lower by the end of the washout period, when H_2_O_2_ production dropped to 86% of normoxic values (Fig. **4d**, *n* = 6 slices from 3 animals, p = 0.0398).

### 3.4 H_2_O_2_ Treatment induces Rundown in ROS Production in Mouse but not NMR Cortex

**Fig. (5):**
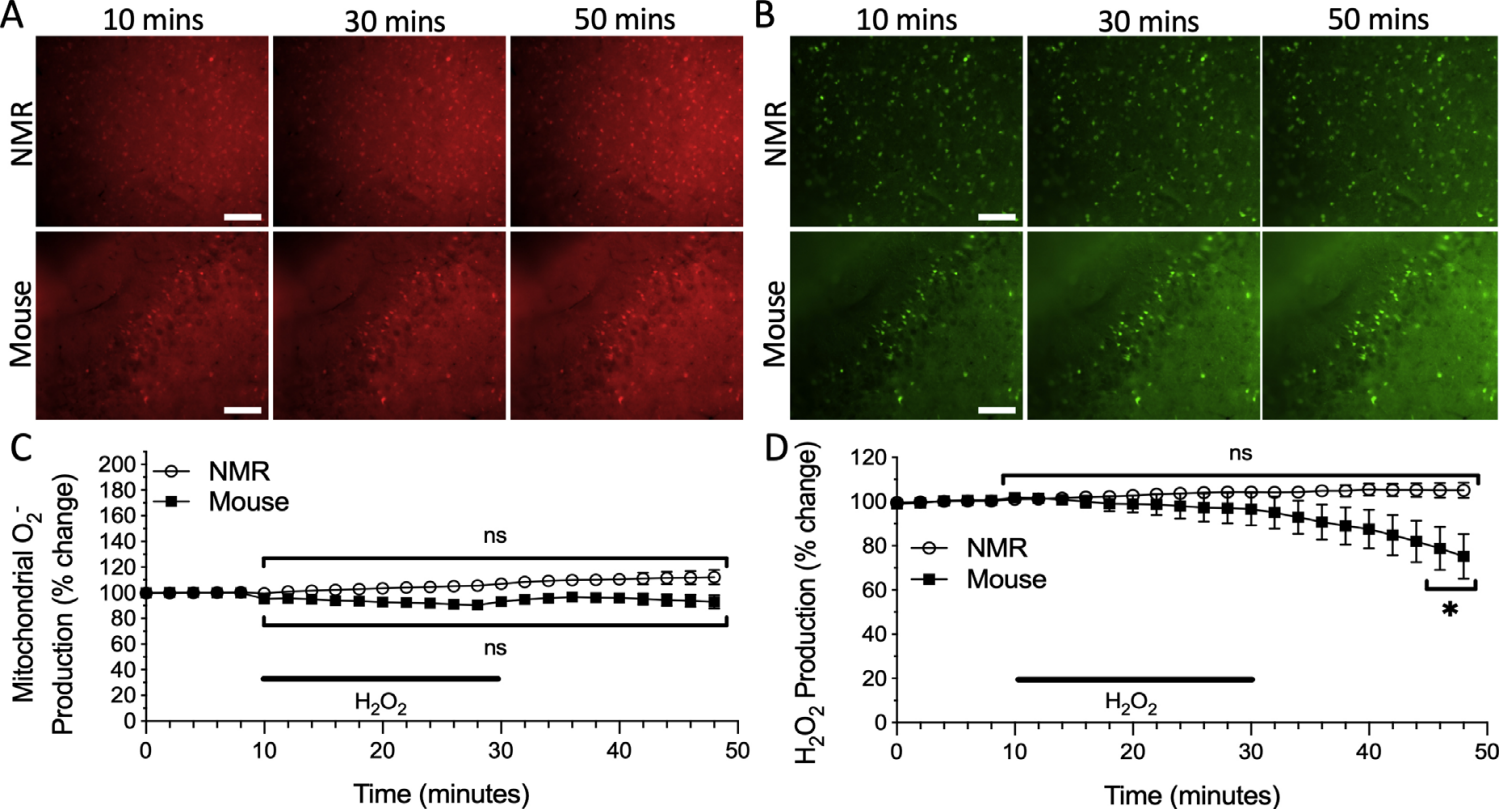
ROS production does not fluctuate during 250 μM H_2_O_2_ exposure in the cortex of mice and NMRs. Fluorescent time-lapse images of cortical slices loaded with **A**) the O_2^•^_^-^ sensitive mitochondrial fluorophore MitoSOX Red, and **B**) the H_2_O_2_ sensitive fluorophore CM-H_2_-DCFDA during initial normoxic conditions, H_2_O_2_ application, and washout. Scale bar = 100 μm. Mean traces of **C**) mitochondrial O_2^•^_^-^ production and **D**) H_2_O_2_ production in the cortex of NMRs (*n* = 5 slices from 4 animals, and 6 slices from 4 animals, respectively) and mice (*n* = 6 slices from 3 animals, for both fluorophores). Individual trials were slope-corrected and normalized based on the initial 10 minutes prior to treatment application. The black bar indicates the H_2_O_2_ treatment application. Asterisk indicates a significant difference in relation to the initial normoxic value (p < 0.05; Two-way ANOVA with Holm-Šídák multiple comparisons test). Data are mean ± SEM.

Finally, we exposed cortical slices to 250 μM H_2_O_2_ to examine the impact of exogenous ROS addition on cellular redox homeostasis. While there was a statistical effect for the interaction between time and species (F_24, 225_ = 2.591, p = 0.0001), application of H_2_O_2_ and subsequent washout had no noticeable effect on mitochondrial O_2^•^_^-^ production over time in either species (Fig. **5b**, *n* = 6 slices from 3 mice and 5 slices from 4 NMRs, F_24, 225_ = 1.190, p = 0.2529).

A significant interaction was observed between time and species with H_2_O_2_ production during H_2_O_2_ treatment trials (F_24, 250_ = 2.078, p = 0.003). Unlike mitochondrial O_2^•^_^-^ production, the application of H_2_O_2_ led to a gradual decrease in H_2_O_2_ production in the mouse cortex, which only became significant towards the end of the washout period where the signal was 76% of its initial value (Fig. **5d**, *n* = 6 slices from 3 animals, p = 0.0059). H_2_O_2_ production in the NMR cortex remained unchanged during H_2_O_2_ application and the following reperfusion (Fig. **5d**, *n* = 6 slices from 4 animals, p > 0.9999).

## 4 DISCUSSION

Using an *in vitro* brain slice model and fluorescence microscopy, we examined changes in ROS homeostasis during and following various hypoxia-related treatment conditions in mouse and NMR cortex as a first step towards developing an *in vitro* brain model to interrogate neuroprotective adaptations against such stresses in a hypoxia-tolerant mammal. Our study has 3 salient findings. First, NMR but not mouse cortex maintains ROS homeostasis at the mitochondrial and whole-cell levels during acute exposure to <1% O_2_, OGD, and during subsequent reoxygenation following both conditions. Second, NMR and mouse cortex do not maintain ROS homeostasis when exposed to chemically induced anoxia. Third, mouse cortex exhibits dysregulation of ROS homeostasis following H_2_O_2_ application while NMR cortex does not, suggesting that NMRs may have an increased ROS scavenging capacity or mechanisms to rapidly down-regulate ROS generation. Together, these findings suggest that NMR cortex maintains ROS homeostasis in both hypoxic and ischemic conditions.

### 4.1 NMRs Maintain Cortical ROS Homeostasis during Acute Hypoxia and Ischemia

**Fig. (6):**
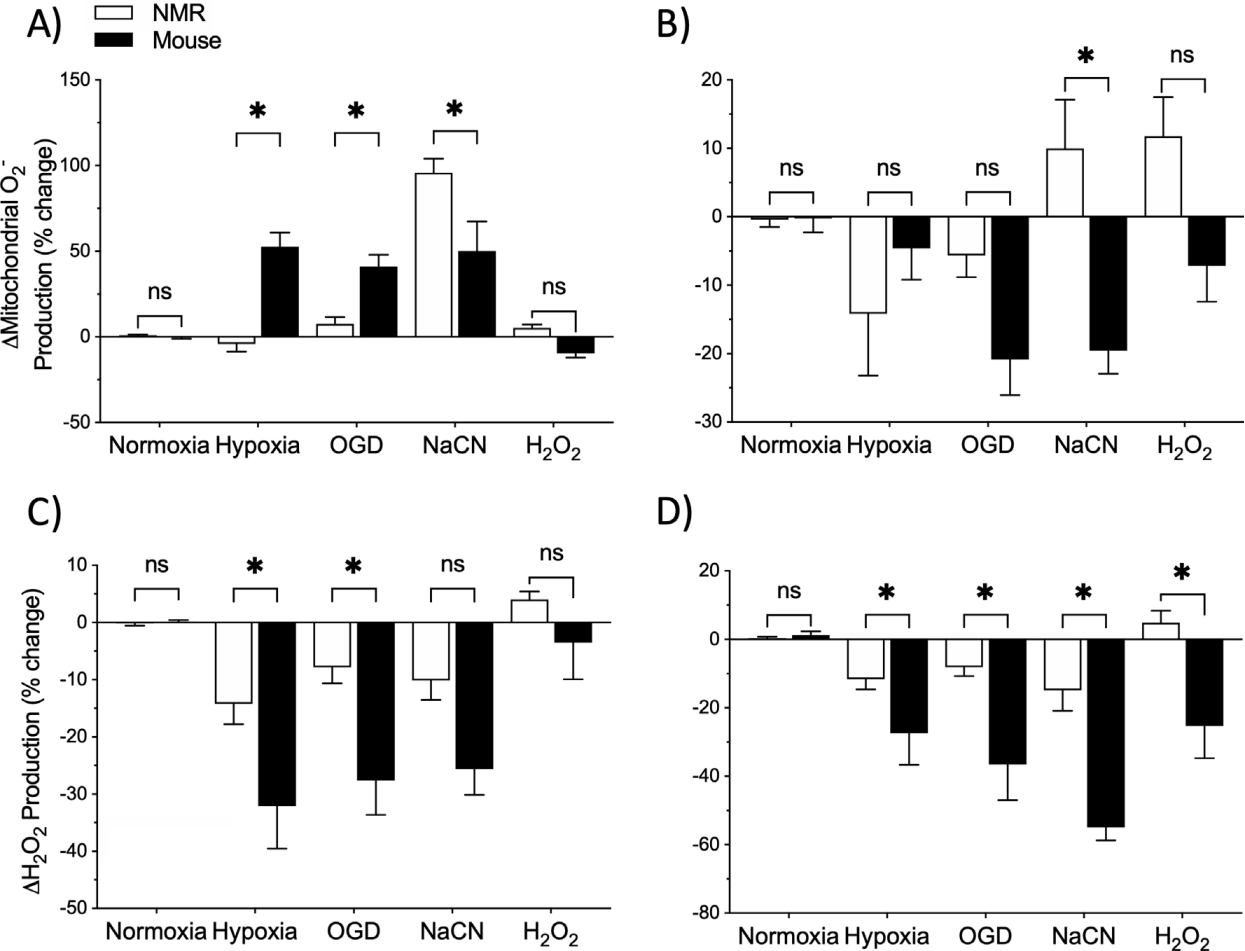
ROS production fluctuates during hypoxia-related treatments and subsequent reperfusion in the cortex of mice, but not NMRs. Changes in mitochondrial O_2^•^_^-^ production from initial normoxic value to **A**) treatment condition and **B**) subsequent re-oxygenation. Changes in H_2_O_2_ production from initial normoxic value to **C**) treatment condition and **D**) subsequent re-oxygenation. Values are differences between mean values within each condition. Asterisk indicate a significant difference in change between species (p < 0.05; Two-way ANOVA with Holm-Šídák multiple comparisons test). Data are mean ± SEM.

Our observations suggest that ROS homeostasis is better maintained in the cortex of a hypoxia-adapted mammal. Specifically, mitochondrial O_2^•^_^-^ and H_2_O_2_ fluctuate during hypoxic and ischemic insults in the mouse cortex, whereas the NMR cortex maintains ROS homeostasis (Fig. **6a**, **c**, p < 0.01). This finding is consistent with other recent studies on the NMR brain in which ROS generation from brain homogenates was lower than in mice following OGD exposure [40], and NO generation from cortical slices was unchanged by acute exposure to hypoxia or OGD [41]. This overall trend observed in NMRs has also been observed in studies investigating other hypoxia tolerant species. For example, after 28-33 days of acclimation to either intermittent (12 hours nocturnal), or constant hypoxia (5 kPa, 2 mg O_2_ l^-1^), ROS emission rates in killifish liver mitochondria decrease by nearly 70% compared to normoxic controls [46]. Similarly, in a strain of hypoxia-adapted (4% O_2_) fruit flies, isolated thorax mitochondria produce 30% and 37% less O_2^•^_^-^ during state 3 and state 4 respiration, respectively, in relation to normoxia-adapted flies [47]. Finally, during more severe anoxic exposures in cortical sheets from western painted turtles (30-minute exposure) and neuronal cultures of pond sliders (4-hour exposure), H_2_O_2_ production decreases by 20% and nearly 100%, respectively, before rapidly returning to initial normoxic levels following reoxygenation in both species [26, 27].

Our findings in mice are also consistent with another study which found that mice are not able to maintain ROS homeostasis during hypoxic challenges. In the mouse cortex, intermittent cyclical exposure to 5.7% O_2_ for 90-second intervals over the course of 1-30 days results in a nearly 2-fold increase in the production of O_2^•^_^-^ and, unlike in our study, H_2_O_2_ as well [48]. Another study similarly observed a nearly 1.7-fold increase in O_2^•^_^-^ production in rat hippocampal and cortical neuron cultures during 40 minutes of OGD (O_2_ < 2 mmHg) [18].

One explanation for these interspecies differences may be that hypoxia-tolerant NMRs better maintain ROS homeostasis during hypoxia/ischemia and/or detoxify ROS to prevent the accumulation of potentially lethal oxidative damage that is otherwise present in hypoxia-intolerant mammals [49–51]. This difference in hypoxic ROS homeostasis may be due to an overall lower rate of ROS production in NMRs that is not impacted by hypoxia or ischemia. There is some evidence to support this hypothesis in other NMR tissues. For example, a recent study from our laboratory found that, after exposure to 7% O_2_ for 4 hours or 11% O_2_ for 4-6 weeks, the rate and capacity of complex I-II ROS efflux in isolated NMR muscle mitochondria are unaffected relative to a 21% O_2_ control group, while complex II-linked H_2_O_2_ efflux decreases by 33% following acute hypoxia [40]. Similarly, a nearly 30% decrease in ROS emission is observed in isolated muscle mitochondria from the hypoxia-tolerant deer mouse following 6-10 weeks of hypobaric hypoxia acclimation [52].

Alternatively, hypoxic ROS production may be similar between mice and NMRs, with the later species instead maintaining ROS homeostasis during hypoxic insults through an improved ROS scavenging capacity that allows them to neutralize free radicals before they can inflict meaningful damage. This is supported by a recent study where, in relation to mice, NMRs were found to have 2- and 5-fold greater capacities to consume H_2_O_2_ by heart and skeletal muscle mitochondria, respectively [43]. Additionally, endogenous antioxidant gene expression in the liver, heart, and brain of two other species of African mole rats (*Spalax galili* and *Spalax judaei*) are elevated compared to rats [53]. Thus, taken together with these previous studies, our data suggest that NMRs may maintain ROS homeostasis during hypoxia through an improved antioxidant capacity and by suppressing ROS production.

### 4.2 NMRs Maintain ROS Homeostasis during Reoxygenation after Hypoxic or Ischemia Exposure

Upon re-oxygenation, NMR mitochondrial O_2^•^_^-^ returns to physiologically normoxical levels, while H_2_O_2_ remains significantly lower in mice compared to NMRs (Fig. **6b**, **d**, p < 0.05). While our results support the idea that NMRs avoid a ROS burst associated with reperfusion injury, our observations in mice do not support previously published research regarding reperfusion injury in that species, particularly as it pertains to succinate-driven reperfusion ROS bursts [21,54]. Considering that complex I is thought to be responsible for elevated neuronal ROS production during both hypoxia and re-oxygenation through a reduced coenzyme Q pool [21,22], it is possible that we do not observe a burst in ROS during reoxygenation in mice because it ROS production is already maximally elevated during hypoxia. The absence of a reoxygenation-induced ROS burst in the NMR cortex is interesting and should be further investigated.

### 4.3 NMRs do not Maintain ROS Homeostasis during Chemically Induced Anoxia

We report that a chemically induced anoxic treatment is the only low-O_2_ challenge that impacts mitochondrial O_2^•^_^-^ in the NMR cortex. The NaCN-mediated upsurge of mitochondrial O_2^•^_^-^ in NMRs is similar in pattern, but significantly greater in magnitude than that observed in mice (Fig. **6a**, p < 0.0001). Conversely, the mouse cortex exhibits a non-significant decrease in H_2_O_2_ compared to NMRs after NaCN (Fig. **6c**, p > 0.05). Interestingly, the continued decrease in H_2_O_2_ following the washout period suggests a loss of function or inability to maintain cellular ROS homeostasis in mice compared to NMRs, despite mitochondrial O_2^•^_^-^ not returning to initial normoxic levels in NMRs following washout (Fig. **6b**, **d**, p < 0.05). These results suggest that NMRs may have an improved ability to maintain cellular ROS homeostasis and a greater capacity for ROS scavenging in the cortex despite compromised mitochondrial ROS homeostasis during chemically-induced anoxia.

Importantly, while NaCN application can be considered chemical anoxia due to its ability to block oxygen from binding to mitochondrial complex IV [18], oxygen is still otherwise available for ROS production from non-mitochondrial sources (*e.g.*, NADPH oxidase and xanthine oxidase), which would not occur during true anoxia. Additionally, NaCN may have indirect effects, including stimulating glutamate release [55], and activation of N-methyl-D-aspartate receptors [56], both of which would trigger excitotoxic cell death cascades mediated by deleterious cytosolic Ca^2+^ accumulation [57], which typically also contributes to derangements in ROS generation. Furthermore, NaCN inhibits superoxide dismutase [58], which could also contribute to the upsurge in mitochondrial O_2^•^_^-^in NMRs.

### 4.4 NMRs Maintain ROS Homeostasis Following Application of Exogenous H_2_O_2_

Unexpectedly, exogenous H_2_O_2_ application does not induce an increase in mitochondrial O_2^•^_^-^or cellular H_2_O_2_ through ROS-induced ROS release [59]. However, much like with NaCN exposure, albeit to a lesser extent, exogenous application of 250 μM H_2_O_2_ does result in a gradual decrease in H_2_O_2_ in the mouse cortex. This fluctuation in ROS is only greater following washout (Fig. **6d**, p < 0.01), but again suggests a loss of function or accumulation of damage in mice that is not present in NMRs. This further supports the idea that, unlike in mice, the NMR cortex maintains ROS homeostasis during oxidative stress by having a greater capacity for ROS scavenging or having a greater tolerance to withstand oxidative stress. These possibilities warrant further investigation.

### 4.5 Implications of Improved Redox Homeostasis in NMR Cortex

There is considerable evidence that disruption of redox homeostasis, including an upsurge in ROS, may result in deleterious oxidative damage to proteins, DNA, and lipids [6, 9-11], resulting in compromised cellular viability and potentially promoting apoptotic cell death [12]. For example, in rats held in simulated hypobaric hypoxic conditions of 6100m above sea level (~10% O_2_) for 3 and 7 days, brain [H_2_O_2_], lipid peroxidation (a marker of oxidative damage), and lactate dehydrogenase (LDH); (an indicator of compromised cell viability) increase by ~30%, ~60%, and ~600%, respectively, following 3 days of hypoxia, and by ~60%, ~300%, and 700%, respectively, after 7 days of hypoxia [60]. Similarly, another study reported a nearly 50% increase in O_2^•^_^-^ production in cultured mouse hippocampal neurons following 2 hours of exposure to an ischemic mimic, which correlates with a 25-fold increase in propidium iodide uptake (a measure of membrane viability) [61]. In a comparative study, LDH release in rat brain rose nearly 5-fold following a 30-minute OGD treatment, whereas LDH only increased 0.5-fold in the brain of the hypoxia-tolerant ground squirrel following the same treatment [62].

Beyond cellular viability, and due to their roles as important signalling molecules, ROS imbalances may also impact other cellular processes, involving protein function, immune response, and ion channel functionality, without completely compromising cellular viability [1-5]. For example, the p62 proteolytic autophagy receptor forms disulphide linked conjugates and leads to activation of autophagy as a result of increased H_2_O_2_ levels, and this is linked to increased cell viability [63]. Severe oxidative stress has also been linked to learning deficits [64] and neurodegenerative disorders, such as Alzheimer’s and Parkinson’s diseases through mechanisms involving mitochondrial dysfunction, transcriptional dysregulation, and dysregulation of calcium signalling [65]. Overall, our results suggest that the NMR cortex has an improved capacity to maintain redox homeostasis during, and following, acute hypoxic exposure compared to mice (Fig. **6**). Taken together with previously published literature, our findings suggest that redox-mediated cell death and derangements in cellular signalling are reduced in NMR brain relative to mouse brain in hypoxia; however, further studies are required to directly test this.

## CONCLUSION

In laboratory experiments, NMRs are able to tolerate harsh hypoxic exposures that are otherwise lethal to mice [29, 32, 33, 66]. Given that imbalances in free radical generation during hypoxia/ischemia and following reperfusion are associated with cell damage, morbidity, and mortality in hypoxia-intolerant animals [11, 12, 67, 68], it is reasonable to speculate that maintaining ROS homeostasis is neuroprotective in this species. Our results support this hypothesis as we report that ROS homeostasis is better maintained in the brain of a hypoxia-adapted mammal. Specifically, we demonstrate that the NMR cortex maintains ROS homeostasis during severe hypoxic insult, ischemia, reperfusion, and application of exogenous ROS, but is unable to do so at the mitochondrial level when exposed to chemical anoxia. These findings set the grounds for further mechanistic investigations on underlying ROS production and scavenging in NMRs, and on the downstream impacts of ROS on cellular viability and signalling. Of particular interest are investigations exploring the regulation of NADPH oxidase, xanthine oxidoreductase, and mitochondrial complexes I and III, all of which are key contributors to ischemia-reperfusion-driven ROS production in mice [18]. Similarly, the ability of the NMR brain to scavenge ROS requires further investigation, as does the impact of maintaining ROS homeostasis on cellular viability in hypoxia and ischemia. Such studies are imperative for expanding our understanding of brain-ischemia pathophysiologies and may additionally yield valuable insights into ways by which we can avoid or prevent hypoxia-induced neurological injury or dysfunction in a mammalian brain.

## AUTHORS’ CONTRIBUTIONS

MEP and LE conceived the idea and designed the study. LE, TW, and MR conducted the experiments and analyzed the data. LE wrote the manuscript and MEP edited it. All authors approved the manuscript in its final form.

## Data Availability

Not applicable.

## LIST OF ABBREVIATIONS

ACSF: Artificial Cerebral Spinal Fluid
CM-H_2_-DCFDA: 6-chloromethyl-2',7'-dichlorodihydrofluorescein diacetate
ETS: Electron Transfer System
H_2_O_2_: Hydrogen Peroxide
NaCN: Sodium Cyanide
NMDG: N-methyl-D-glucamine
NMR: Naked Mole-rat
O_2^•^_^-^: Superoxide
OGD: Oxygen Glucose Deprivation
ROS: Reactive Oxygen Species

## References

[1] Wang Y., Zang Q.S., Liu Z., Wu Q., Maass D., Dulan G., Shaul P.W., Melito L., Frantz D.E., Kilgore J.A., Williams N.S., Terada L.S., Nwariaku F.E. (2011). Regulation of VEGF-induced endothelial cell migration by mitochondrial reactive oxygen species.. Am. J. Physiol. Cell Physiol..

[2] West A.P., Shadel G.S., Ghosh S. (2011). Mitochondria in innate immune responses.. Nat. Rev. Immunol..

[3] Finkel T. (2012). Signal transduction by mitochondrial oxidants.. J. Biol. Chem..

[4] Kiselyov K., Muallem S. (2016). ROS and intracellular ion channels.. Cell Calcium.

[5] Scherz-Shouval R., Shvets E., Fass E., Shorer H., Gil L., Elazar Z. (2007). Reactive oxygen species are essential for autophagy and specifically regulate the activity of Atg4.. EMBO J..

[6] Starkov A.A., Andreyev A.Y., Zhang S.F., Starkova N.N., Korneeva M., Syromyatnikov M., Popov V.N. (2014). Scavenging of H2O2 by mouse brain mitochondria.. J. Bioenerg. Biomembr..

[7] Munro D., Banh S., Sotiri E., Tamanna N., Treberg J.R. (2016). The thioredoxin and glutathione-dependent H2O2 consumption pathways in muscle mitochondria: Involvement in H2O2 metabolism and consequence to H2O2 efflux assays.. Free Radic. Biol. Med..

[8] Drechsel D.A., Patel M. (2010). Respiration-dependent H2O2 removal in brain mitochondria via the thioredoxin/peroxiredoxin system.. J. Biol. Chem..

[9] Martínez M.C., Andriantsitohaina R. (2009). Reactive nitrogen species: Molecular mechanisms and potential significance in health and disease.. Antioxid. Redox Signal..

[10] Apel K., Hirt H. (2004). Reactive oxygen species: Metabolism, oxidative stress, and signal transduction.. Annu. Rev. Plant Biol..

[11] Levraut J., Iwase H., Shao Z.H., Vanden Hoek T.L., Schumacker P.T. (2003). Cell death during ischemia: Relationship to mitochondrial depolarization and ROS generation.. Am. J. Physiol. -Hear. Circ. Physiol..

[12] Marchi S., Giorgi C., Suski J.M., Agnoletto C., Bononi A., Bonora M., De Marchi E., Missiroli S., Patergnani S., Poletti F., Rimessi A., Duszynski J., Wieckowski M.R., Pinton P. (2012). Mitochondria-ros crosstalk in the control of cell death and aging.. J. Signal Transduct..

[13] Adam-Vizi V. (2005). Production of reactive oxygen species in brain mitochondria: Contribution by electron transport chain and non-electron transport chain sources.. Antioxid. Redox Signal..

[14] Chandel N.S., Maltepe E., Goldwasser E., Mathieu C.E., Simon M.C., Schumacker P.T. (1998). Mitochondrial reactive oxygen species trigger hypoxia-induced transcription.. Proc. Natl. Acad. Sci. USA.

[15] Berry C.E., Hare J.M. (2004). Xanthine oxidoreductase and cardiovascular disease: Molecular mechanisms and pathophysiological implications.. J. Physiol..

[16] Guzy R.D., Hoyos B., Robin E., Chen H., Liu L., Mansfield K.D., Simon M.C., Hammerling U., Schumacker P.T. (2005). Mitochondrial complex III is required for hypoxia-induced ROS production and cellular oxygen sensing.. Cell Metab..

[17] MacGregor D.G., Avshalumov M.V., Rice M.E. (2003). Brain edema induced by in vitro ischemia: Causal factors and neuroprotection.. J. Neurochem..

[18] Abramov A.Y., Scorziello A., Duchen M.R. (2007). Three distinct mechanisms generate oxygen free radicals in neurons and contribute to cell death during anoxia and reoxygenation.. J. Neurosci..

[19] Fekete A., Vizi E.S., Kovács K.J., Lendvai B., Zelles T. (2008). Layer-specific differences in reactive oxygen species levels after oxygen-glucose deprivation in acute hippocampal slices.. Free Radic. Biol. Med..

[20] Wilson D.F., Rumsey W.L., Green T.J., Vanderkooi J.M. (1988). The oxygen dependence of mitochondrial oxidative phosphorylation measured by a new optical method for measuring oxygen concentration.. J. Biol. Chem..

[21] Scialò F., Fernández-Ayala D.J., Sanz A. (2017). Role of mitochondrial reverse electron transport in ROS signaling: Potential roles in health and disease.. Front. Physiol..

[22] Orr A.L., Ashok D., Sarantos M.R., Shi T., Hughes R.E., Brand M.D. (2013). Inhibitors of ROS production by the ubiquinone-binding site of mitochondrial complex I identified by chemical screening.. Free Radic. Biol. Med..

[23] Quinlan C.L., Orr A.L., Perevoshchikova I.V., Treberg J.R., Ackrell B.A., Brand M.D. (2012). Mitochondrial complex II can generate reactive oxygen species at high rates in both the forward and reverse reactions.. J. Biol. Chem..

[24] Paddenberg R., Ishaq B., Goldenberg A., Faulhammer P., Rose F., Weissmann N., Braun-Dullaeus R. C., Kummer W. (2003). Essential role of complex II of the respiratory chain in hypoxia-induced ROS generation in the pulmonary vasculature.. Am. J. Physiol. - Lung Cell. Mol. Physiol..

[25] Du G., Mouithys-Mickalad A., Sluse F.E. (1998). Generation of superoxide anion by mitochondria and impairment of their functions during anoxia and reoxygenation in vitro.. Free Radic. Biol. Med..

[26] Milton S.L., Nayak G., Kesaraju S., Kara L., Prentice H.M. (2007). Suppression of reactive oxygen species production enhances neuronal survival in vitro and in vivo in the anoxia-tolerant turtle Trachemys scripta.. J. Neurochem..

[27] Pamenter M.E., Richards M.D., Buck L.T. (2007). Anoxia-induced changes in reactive oxygen species and cyclic nucleotides in the painted turtle.. J. Comp. Physiol. B.

[28] Hogg D.W., Pamenter M.E., Dukoff D.J., Buck L.T. (2015). Decreases in mitochondrial reactive oxygen species initiate GABA(A) receptor-mediated electrical suppression in anoxia-tolerant turtle neurons.. J. Physiol..

[29] Larson J., Park T.J. (2009). Extreme hypoxia tolerance of naked mole-rat brain.. Neuroreport.

[30] Braude S., Holtze S., Begall S., Brenmoehl J., Burda H., Dammann P., Del Marmol D., Gorshkova E., Henning Y., Hoeflich A., Höhn A., Jung T., Hamo D., Sahm A., Shebzukhov Y., Šumbera R., Miwa S., Vyssokikh M.Y., von Zglinicki T., Averina O., Hildebrandt T.B. (2021). Surprisingly long survival of premature conclusions about naked mole-rat biology.. Biol. Rev. Camb. Philos. Soc..

[31] Buffenstein R., Amoroso V., Andziak B., Avdieiev S., Azpurua J., Barker A.J., Bennett N.C., Brieño Enríquez M.A., Bronner G.N., Coen C., Delaney M.A., Dengler Crish C.M., Edrey Y.H., Faulkes C.G., Frankel D., Friedlander G., Gibney P.A., Gorbunova V., Hine C., Holmes M.M., Jarvis J.U.M., Kawamura Y., Kutsukake N., Kenyon C., Khaled W.T., Kikusui T., Kissil J., Lagestee S., Larson J., Lauer A., Lavrenchenko L.A., Lee A., Levitt J.B., Lewin G.R., Lewis H.K.N., Lin T.D., Mason M.J., McCloskey D., McMahon M., Miura K., Mogi K., Narayan V., O’Connor T.P., Okanoya K., O’Riain M.J., Park T.J., Place N.J., Podshivalova K., Pamenter M.E., Pyott S.J., Reznick J., Ruby J.G., Salmon A.B., Santos Sacchi J., Sarko D.K., Seluanov A., Shepard A., Smith M., Storey K.B., Tian X., Vice E.N., Viltard M., Watarai A., Wywial E., Yamakawa M., Zemlemerova E.D., Zions M., Smith E.S.J. (2002). The naked truth: A comprehensive clarification and classification of current ‘Myths’ in naked mole rat biology.. Biol. Rev. Camb. Philos. Soc..

[32] Ilacqua A.N., Kirby A.M., Pamenter M.E. (2017). Behavioural responses of naked mole rats to acute hypoxia and anoxia.. Biol. Lett..

[33] Kirby A.M., Fairman G.D., Pamenter M.E. (2018). Atypical behavioural, metabolic and thermoregulatory responses to hypoxia in the naked mole rat (heterocephalus glaber).. J. Zool. (Lond.).

[34] Park T.J., Reznick J., Peterson B.L., Blass G., Omerbašić D., Bennett N.C., Kuich P.H.J.L., Zasada C., Browe B.M., Hamann W., Applegate D.T., Radke M.H., Kosten T., Lutermann H., Gavaghan V., Eigenbrod O., Bégay V., Amoroso V.G., Govind V, Minshall R.D., Smith E.S.J., Larson J., Gotthardt M., Kempa S., Lewin G.R. (2017). Fructose-driven glycolysis supports anoxia resistance in the naked mole-rat.. Science (80-.).

[35] Peterson B.L., Larson J., Buffenstein R., Park T.J., Fall C.P. (2012). Blunted neuronal calcium response to hypoxia in naked mole-rat hippocampus.. PLoS One.

[36] Pamenter M.E., Lau G.Y., Richards J.G., Milsom W.K. (2018). Naked mole rat brain mitochondria electron transport system flux and H+ leak are reduced during acute hypoxia.. J. Exp. Biol..

[37] Farhat E., Devereaux M.E.M., Cheng H., Weber J.M., Pamenter M.E. (2021). Na+/K+-ATPase activity is regionally regulated by acute hypoxia in naked mole-rat brain.. Neurosci. Lett..

[38] Cheng H., Qin Y.A., Dhillon R., Dowell J., Denu J.M., Pamenter M.E. (2022). Metabolomic analysis of carbohydrate and amino acid changes induced by hypoxia in naked mole-rat brain and liver.. Metabolites.

[39] Pamenter M.E., Dzal Y.A., Thompson W.A., Milsom W.K. (2019). Do naked mole rats accumulate a metabolic acidosis or an oxygen debt in severe hypoxia?. J. Exp. Biol..

[40] Cheng H., Munro D., Huynh K., Pamenter M.E. (2021). Naked mole-rat skeletal muscle mitochondria exhibit minimal functional plasticity in acute or chronic hypoxia.. Comp. Biochem. Physiol. B Biochem. Mol. Biol..

[41] Wang T.H., Eaton L., Pamenter M.E. (2020). Nitric oxide homeostasis is maintained during acute in vitro hypoxia and following reoxygenation in naked mole-rat but not mouse cortical neurons.. Comp. Biochem. Physiol. A Mol. Integr. Physiol..

[42] Cheng H., Pamenter M.E. (2021). Naked mole-rat brain mitochondria tolerate in vitro ischaemia.. J. Physiol..

[43] Munro D., Baldy C., Pamenter M.E., Treberg J.R. (2019). The exceptional longevity of the naked mole-rat may be explained by mitochondrial antioxidant defenses.. Aging Cell.

[44] Munro D., Pamenter M.E. (2019). Comparative studies of mitochondrial reactive oxygen species in animal longevity: Technical pitfalls and possibilities.. Aging Cell.

[45] Lesuisse C., Martin L.J. (2002). Long-term culture of mouse cortical neurons as a model for neuronal development, aging, and death.. J. Neurobiol..

[46] Du S.N.N., Mahalingam S., Borowiec B.G., Scott G.R. (2016). Mitochondrial physiology and reactive oxygen species production are altered by hypoxia acclimation in killifish (Fundulus heteroclitus).. J. Exp. Biol..

[47] Ali S.S., Hsiao M., Zhao H.W., Dugan L.L., Haddad G.G., Zhou D. (2012). Hypoxia-adaptation involves mitochondrial metabolic depression and decreased ROS leakage.. PLoS One.

[48] Xu W., Chi L., Row B.W., Xu R., Ke Y., Xu B., Luo C., Kheirandish L., Gozal D., Liu R. (2004). Increased oxidative stress is associated with chronic intermittent hypoxia-mediated brain cortical neuronal cell apoptosis in a mouse model of sleep apnea.. Neuroscience.

[49] Auten R.L., Davis J.M. (2009). Oxygen toxicity and reactive oxygen species: The devil is in the details.. Pediatr. Res..

[50] Schieber M., Chandel N.S. (2014). ROS function in redox signaling and oxidative stress.. Curr. Biol..

[51] Torres-Cuevas I., Corral-Debrinski M., Gressens P. (2019). Brain oxidative damage in murine models of neonatal hypoxia/ischemia and reoxygenation.. Free Radic. Biol. Med..

[52] Mahalingam S., McClelland G.B., Scott G.R. (2017). Evolved changes in the intracellular distribution and physiology of muscle mitochondria in high-altitude native deer mice.. J. Physiol..

[53] Schülke S., Dreidax D., Malik A., Burmester T., Nevo E., Band M., Avivi A., Hankeln T. (2012). Living with stress: Regulation of antioxidant defense genes in the subterranean, hypoxia-tolerant mole rat,. Spalax. Gene.

[54] Chouchani E.T., Pell V.R., Gaude E., Aksentijević D., Sundier S.Y., Robb E.L., Logan A., Nadtochiy S.M., Ord E.N.J., Smith A.C., Eyassu F., Shirley R., Hu C.H., Dare A.J., James A.M., Rogatti S., Hartley R.C., Eaton S., Costa A.S.H., Brookes P.S., Davidson S.M., Duchen M.R., Saeb-Parsy K., Shattock M.J., Robinson A.J., Work L.M., Frezza C., Krieg T., Murphy M.P. (2014). Ischaemic accumulation of succinate controls reperfusion injury through mitochondrial ROS.. Nature.

[55] Dong Y., Zhang W., Lai B., Luan W.J., Zhu Y.H., Zhao B.Q., Zheng P. (2012). Two free radical pathways mediate chemical hypoxia-induced glutamate release in synaptosomes from the prefrontal cortex.. Biochim. Biophys. Acta.

[56] Jensen M.S., Ahlemeyer B., Ravati A., Thakur P., Mennel H.D., Krieglstein J. (2002). Preconditioning-induced protection against cyanide-induced neurotoxicity is mediated by preserving mitochondrial function.. Neurochem. Int..

[57] Choi D.W. (1992). Excitotoxic cell death.. J. Neurobiol..

[58] Ozaki S., Hirose J., Kidani Y. (1988). Electron-transfer reaction between Fe(CN)64-/Fe(CN)63- and Copper(II)/Copper(I) ions in bovine erythrocyte superoxide dismutase: Ph dependence and inhibition by various kinds of anions.. Inorg. Chem..

[59] Zorov D.B., Juhaszova M., Sollott S.J. (2014). Mitochondrial reactive oxygen species (ROS) and ROS-induced ROS release.. Physiol. Rev..

[60] Maiti P., Singh S.B., Sharma A.K., Muthuraju S., Banerjee P.K., Ilavazhagan G. (2006). Hypobaric hypoxia induces oxidative stress in rat brain.. Neurochem. Int..

[61] Pamenter M.E., Ali S.S., Tang Q., Finley J.C., Gu X.Q., Dugan L.L., Haddad G.G. (2012). An in vitro ischemic penumbral mimic perfusate increases NADPH oxidase-mediated superoxide production in cultured hippocampal neurons.. Brain Res..

[62] Bhowmick S., Moore J.T., Kirschner D.L., Drew K.L. (2017). Arctic ground squirrel hippocampus tolerates oxygen glucose deprivation independent of hibernation season even when not hibernating and after ATP depletion, acidosis, and glutamate efflux.. J. Neurochem..

[63] Carroll B., Otten E.G., Manni D., Stefanatos R., Menzies F.M., Smith G.R., Jurk D., Kenneth N., Wilkinson S., Passos J.F., Attems J., Veal E.A., Teyssou E., Seilhean D., Millecamps S., Eskelinen E.L., Bronowska A.K., Rubinsztein D.C., Sanz A., Korolchuk V.I. (2018). Oxidation of SQSTM1/p62 mediates the link between redox state and protein homeostasis.. Nat. Commun..

[64] Row B.W., Liu R., Xu W., Kheirandish L., Gozal D. (2003). Intermittent hypoxia is associated with oxidative stress and spatial learning deficits in the rat.. Am. J. Respir. Crit. Care Med..

[65] Sbodio J.I., Snyder S.H., Paul B.D. (2019). Redox mechanisms in neurodegeneration: From disease outcomes to therapeutic opportunities.. Antioxid. Redox Signal..

[66] Døhlen G., Carlsen H., Blomhoff R., Thaulow E., Saugstad O.D. (2005). Reoxygenation of hypoxic mice with 100% oxygen induces brain nuclear factor-kappa B.. Pediatr. Res..

[67] Dhar-Mascareño M., Cárcamo J.M., Golde D.W. (2005). Hypoxia-reoxygenation-induced mitochondrial damage and apoptosis in human endothelial cells are inhibited by vitamin C.. Free Radic. Biol. Med..

[68] Chouchani E.T., Pell V.R., James A.M., Work L.M., Saeb-Parsy K., Frezza C., Krieg T., Murphy M.P. (2016). A unifying mechanism for mitochondrial superoxide production during ischemiareperfusion injury.. Cell Metab..

